# Effects of the adenosinergic system on the expression and acquisition of sensitization to conditioned place preference in morphine-conditioned rats

**DOI:** 10.1007/s00210-015-1190-6

**Published:** 2015-12-05

**Authors:** Joanna Listos, Sylwia Talarek, Piotr Listos, Jolanta Orzelska, Małgorzata Łupina, Sylwia Fidecka

**Affiliations:** Department of Pharmacology and Pharmacodynamics, Medical University of Lublin, Chodźki 4a St., 20-093 Lublin, Poland; Department of Pathological Anatomy, Faculty of Veterinary Medicine, University of Life Sciences, Głęboka 30 St., 20-612 Lublin, Poland

**Keywords:** Adenosine receptor agonists and antagonists, Behavioral sensitization, Conditioned place preference, Priming dose

## Abstract

In the presented study, we attempt to investigate if the sensitization to conditioned place preference (CPP) induced by low doses of morphine was developed in rats which have been previously conditioned with morphine. The experiments were performed in the CPP test. Firstly, it has been demonstrated that administration of ineffective dose of morphine on the 9th day induces the increase in time spent of rats at a morphine-paired compartment, confirming that sensitization to CPP has been developed in these animals. Secondly, it has been shown that stimulation of A_1_ receptor significantly inhibits the expression of morphine-induced of sensitization, and blockade of these receptors produces the opposite effect. Finally, it has been indicated that both stimulation and blockade of A_1_ and/or A_2A_ receptors inhibit the acquisition of sensitization to CPP. The obtained results have strongly supported the significance of adenosinergic system in both expression and acquisition of studied sensitization. These results seem to be important for the identification of connections in the central nervous system which can help finding new strategies to attenuate rewarding action of morphine.

## Introduction

Adenosine, a potent inhibitory neuromodulator in the central nervous system, acts via four most recognized adenosine receptor subtypes: A_1_, A_2A_, A_2B_, and A_3_. A_1_ receptors are highly expressed in different brain areas, such as the cortex, the cerebellum, the hippocampus, and dorsal horn of the spinal cord. The distribution of A_2A_ receptors is more limited and found mainly in the striatopallidal γ-aminobutyric acid (GABA)ergic neurons and in the olfactory bulb, while in other brain areas, the receptors are expressed at lower levels (Ferré et al. 1997). As a neuromodulator of the central nervous system, adenosine may influence different processes, like sleep, cognition, pain, etc. Many experimental data also confirm adenosine participation in the state of dependence. As literature data have already shown, the significance of adenosine receptors has repeatedly been demonstrated in various models of behavioral sensitization, expressed by locomotor responses. For example, a stimulation of A_2A_ receptors significantly inhibited both the expression and acquisition of cocaine-induced sensitization in rats (Filip et al. 2006). the expression of cocaine-induced sensitization was also inhibited after stimulation of A_1_ or A_2A_ receptors in the nucleus accumbens of rats (Hobson et al. 2012). Additionally, Shimazoe et al. (2000) have shown the importance of A_1_ and A_2A_ receptors in both the expression and acquisition of methamphetamine-induced sensitization in rats. Similarly, in our previous study, an involvement of adenosine A_1_ and/or A_2A_ receptors was demonstrated in the acquisition of morphine-induced behavioral sensitization to the locomotor activity in mice (Listos et al. 2011).

Behavioral sensitization is defined as long-lasting and progressive enhancement of the locomotor and motivational responses to psychostimulants, following their repeated and intermittent administration. The sensitized animals begin to develop addiction-like symptoms, including continued drug-seeking behavior and escalated drug intake, an increased motivation to find or receive drugs and a greater propensity to relapse after enforced abstinence (Robinson and Berridge 2008). This phenomenon plays an important role in the etiology and maintenance of drug-seeking behavior, as well as in relapse to drug use, even after long-term abstinence periods (Robinson and Berridge 1993, 2000; Stewart and Badiani 1993). Behavioral sensitization is the subject of numerous investigations, attempted to identify drug relapse mechanisms and design new therapeutic strategies, effective in drug addiction treatment. Sensitization can be estimated in various animal models, mainly in the locomotor activity test (Harris et al. 2014; Listos et al. 2011; Martinez et al. 2014). In this paradigm, the administration of a psychoactive drug in a low dose (the priming dose) after a few days of interruption in the drug use significantly intensifies the locomotor activity of animals, confirming that drug-seeking behaviors have been developed. Although studies on behavioral sensitization are focused on increased locomotor activity of animals, a growing body of evidence shows that sensitization may be developed to other types of influence. For example, hyperalgesia (Ahmadi et al. 2014) or higher sensitivity to morphine-induced place preference (Sahraei et al. 2007) was observed in morphine-sensitized mice. Other authors also demonstrated in the conditioned place preference (CPP) paradigm that a previous exposure to morphine produced enhanced rewarding effects after morphine intake (Lett 1989; Manzanedo et al. 2005; Shippenberg et al 1998). Additionally, Manzanedo et al. (2005) demonstrated some involvement of dopaminergic system in this phenomenon. Thus, although most studies on behavioral sensitization in animals are concentrated on their increased locomotor activity, it seems both important and interesting to extend our knowledge on sensitization to other effects.

Therefore, in the presented study, we attempt to investigate if sensitization to conditioned place preference (CPP), induced by low doses of morphine, was developed in rats which had previously been conditioned with morphine. The experiments were performed in the CPP test. This test consists of three phases: pre-conditioning, conditioning, and post-conditioning, and is generally accepted in preclinical studies for assessment of the rewarding properties of various abused drugs (Huston et al. 2013; Lett 1989; Manzanedo et al. 2005; Shippenberg et al 1998). In order to develop sensitization to CPP, after the post-conditioning stage, four test-free days followed, and on the 9th day of the experiment, the post-conditioning stage with the priming dose of morphine was carried out afresh. Furthermore, effects of adenosine A_1_ and A_2A_ ligands on the expression and acquisition of morphine-induced sensitization to CPP in rats were studied in the presented study. Regarding the expression, acute effects of adenosine ligands were studied in morphine-induced sensitization to CPP, while in acquisition, the significance of longer exposure (three injections) of adenosinergic agents was investigated during the development of behavioral sensitization. In our previous study (Listos et al. 2011). we showed that adenosine agonists significantly attenuated morphine-induced sensitization to the locomotor activity of mice, which reflected morphine-seeking behavior. In another study, we demonstrated that adenosine agonists (2-p-(2-carboxyethyl)phenethylamino-5′-N-ethylcarboxamidoadenosine hydrochloride (CGS 21680) and 5′-N-ethylcarboxamidoadenosine (NECA)) were able to inhibit the development of hypersensitivity to acute dose of morphine given during morphine withdrawal period (Listos et al. 2008). which also reflected morphine-seeking behavior. Now, we present experiments which show the role of both adenosine agonists and antagonists, in sensitization to CPP, which reflect the sensitization to rewarding action of morphine. As it has been mentioned, the adenosinergic system plays an important role in different aspects of behavioral sensitization; thus, it is worth recognizing its involvement in sensitization to CPP in rats.

## Material and methods

### Animals

The experiments were carried out on male Wistar rats (250–300 g). The animals were kept at room temperature of 22 ± 1 °C, on natural day–night cycle. Standard food (Murigran pellets, Bacutil, Motycz) and tap water were freely available. All the experiments were made between 9 a.m. and 3 p.m. After one week of adaptation and handling, the animals were divided into groups (9–10 animals/group) and prepared for the tests.

The study was performed according to the National Institute of Health Guidelines for the Care and Use of Laboratory Animals and the European Community Council Directive for Care and Use of Laboratory Animals and was approved by local ethics committee (the Medical University of Lublin Committee on the Use and Care of Animals, No. 45/2007).

### Drugs

The following drugs were used in the experiments: morphine hydrochloride (Polfa, Kutno, Poland) and adenosine receptor ligands: N^6^-cyclopentyladenosine (CPA)—the selective adenosine A_1_ receptor agonist; 2-p-(2-carboxyethyl)phenethylamino-5′-N-ethylcarboxamidoadenosine hydrochloride (CGS 21680)—the selective adenosine A_2A_ receptor agonist; 5′-N-ethylcarboxamidoadenosine (NECA)—the non-selective adenosine A_1_/A_2A_ receptor agonist with low affinity for A_2B_ and A_3_ receptors; 8-cyclopentyl-1,3-dipropylxanthine (DPCPX)––the adenosine A_1_ receptor antagonist with low affinity for A_2B_ receptors; 2-(2-furanyl)–7-(2-phenylethyl)-7*H*-pyrazolo(4,3-e)(1,2,4)triazolo(1,5-c)pyrimidin-5-amine (SCH 58261)—the selective adenosine A_2A_ receptor antagonist—(all from Sigma-Aldrich, St. Louis, USA), and caffeine—the non-selective adenosine A_1_/A_2A_ receptor antagonist (Polfa, Kutno, Poland).

CPA, CGS 21680, caffeine, and morphine were dissolved in saline; NECA, DPCPX, and SCH 58261 were dissolved in minimal volume of DMSO (final concentration 0.1 %); and then they were diluted in saline.

All drugs were given intraperitoneally (ip) in a volume of 10.0 ml/kg.

The following doses of drugs were used in the experiments: morphine: 5.0 mg/kg—for conditioning or 0.5 and 0.75 mg/kg as a priming dose for sensitization, CPA: 0.05 and 0.1 mg/kg, CGS 21680: 0.025 and 0.05 mg/kg, NECA: 0.0005 and 0.001 mg/kg, DPCPX: 1.0 and 2.0 mg/kg, SCH 58261: 0.5 and 1.0 mg/kg, and caffeine: 5.0 and 10.0 mg/kg. The control group animals received the same volume of saline at the respective time before the test.

### Apparatus and procedure

In all experiments, the CPP apparatus was used. The applied equipment consisted of eight rectangular boxes (60 × 35 × 30 cm), each of them divided into three compartments (25 × 30 cm), separated by removable guillotine doors from a small central gray area (10 × 10 cm). The walls of the two large compartments differed in color (black floors and walls in one compartment and white floors and walls in the other one). The boxes were kept in a soundproof room with a neutral masking noise and with a dim 40 lx illumination. The animal behavior was observed on a monitor, displaying images from a digital video camera system, while the time periods, spent by rats in each compartment, were recorded by means of video tracking software (Karnet, Lublin, Poland).

The procedure of the experiments was based on the protocol, described by Sahraei et al. (2007). that consisted of three, typical for CPP paradigm, phases: pre-conditioning, conditioning, and post-conditioning. Four days after the post-conditioning phase (it means, five days after discontinuation of morphine treatment), the induction of sensitization to the CPP in rats was performed.

Pre-conditioning (the 1st day): This day, each rat was placed separately into the central gray area for 15 min (900 s) and left with free access to all the compartments. The time period spent by each animal in the two large compartments was measured and recorded.

Conditioning (the 2nd–4th day): That phase consisted of a 3-day schedule of conditioning sessions. Each day, two sessions were performed. During the first session, the rats received saline and were placed in the preferred (black) compartment—morning sessions—for 30 min. Next, the rats received morphine (5.0 mg/kg) and were placed in the unpreferred compartment for the same period of time—afternoon sessions. The intervals between saline and morphine injections were at least 6 h. The procedure was repeated on the 2nd and the 3rd days of conditioning.

Post-conditioning (the 5th day): On the 5th day (the preference test day), similar to the pre-conditioning phase, the animals were placed into the central gray area for 15 min (900 s), and the time period spent in the morphine-paired compartment was recorded for each animal. No injections were given on that day.

Induction of sensitization (the 9th day) to CPP in previously morphine-conditioned rats: On the 9th day of the experiment (four days after the post-conditioning = five days after the last morphine injection) to induce sensitization to CPP, an ineffective dose of morphine (0.5 or 0.75 mg/kg)—the priming dose—was injected, and then, the post-conditioning paradigm was performed once again.

**Scheme 1:**
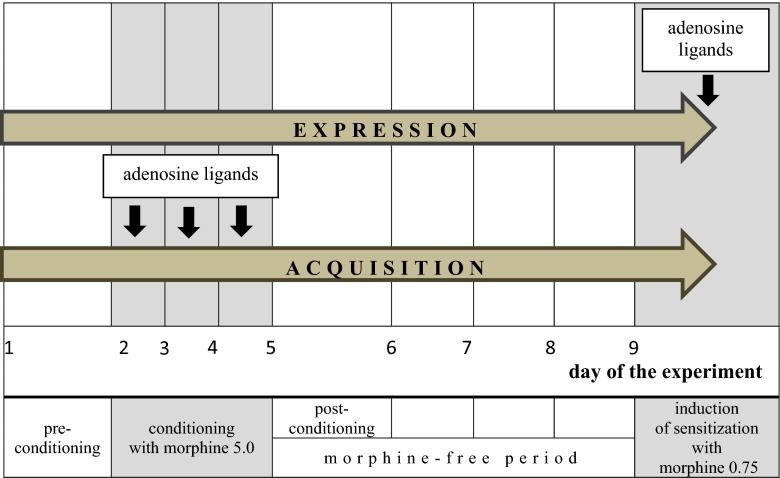
The method of administration of adenosine ligands in expression and acquisition of sensitization to CPP

Effects of the adenosinergic system on sensitization to CPP induced by priming dose of morphine: In order to evaluate the influence of adenosine receptors on the expression of sensitization to CPP, on the 9th day of the experiment, adenosine agonists or antagonists were administered 20 min before the ineffective dose of morphine. In order to assess the effect of adenosine ligands on the acquisition of sensitization to CPP, all the adenosine drugs were injected three times on the 2nd–4th day, 20 min before each morphine injection (5.0 mg/kg). The method of administration of adenosine ligands is shown in Scheme 1.

### Statistical analysis

The obtained data are presented in the figures as the mean values ± S.E.M of scores. Score is a difference in the time period [s] which the rats spent at a morphine-paired compartment. It was calculated according to the following formula:

- for evaluation of behavioral sensitization: time period at the morphine-paired compartment on the 9th day minus the time period at the morphine-paired compartment on the 1st day, and
- for evaluation of conditioning: time period at the morphine-paired compartment on the 5th day minus time period at the morphine-paired compartment on the 1st day.

The locomotor activity of individual rats was measured as the total distance, traveled during 15-min (900 s) period.

All the results were statistically calculated using the Graph Pad Prism Software Package (version 5.04).

**Fig. 1:**
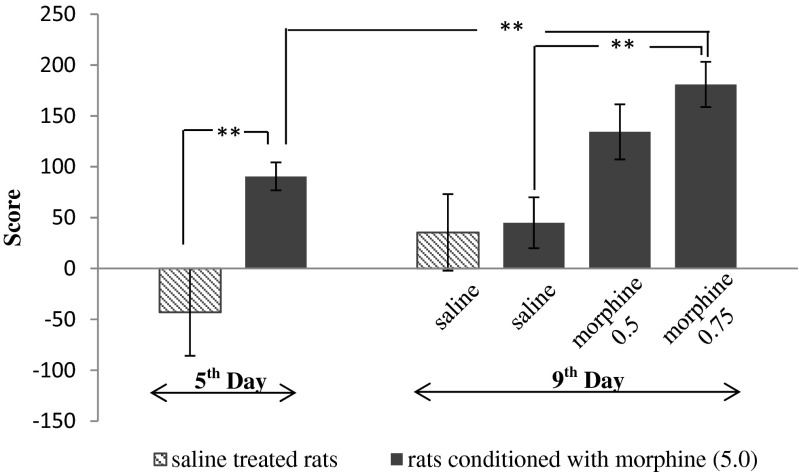
Influence of ineffective morphine doses on morphine-conditioned rats (5 mg/kg, ip). In order to morphine-induce sensitization to CPP, its ineffective doses (0.5 and 0.75 mg/kg, ip) were administered on 9th day. Results are expressed as mean ± S.E.M of scores (*n* = 9–10 rats/group). Two-way ANOVA showed significant changes in the time spent at the morphine-paired compartment. **p < 0.01 (Tukey test)

**Fig. 2:**
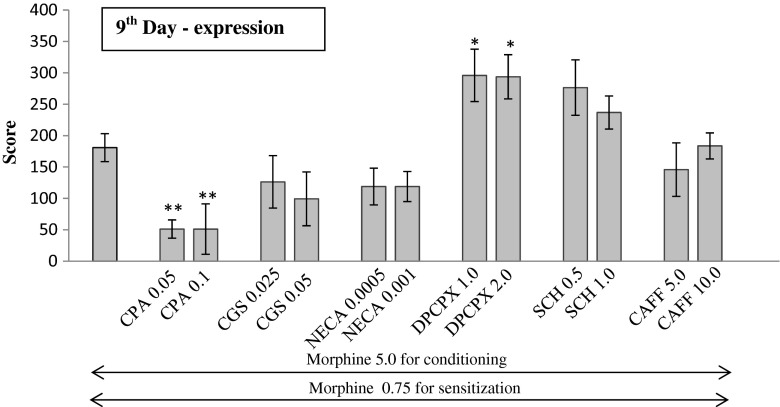
Influence of adenosine agonists and antagonists on the expression of sensitization to CPP in morphine-conditioned rats. Morphine sensitization was obtained by administration of priming dose of morphine (0.75 mg/kg, ip) in morphine-conditioned rats (5 mg/kg, ip). Adenosine ligands were injected 20 min before the priming dose of morphine (on 9th day). Results are expressed as mean ± S.E.M of scores (*n* = 9–10 rats/group). One-way ANOVA showed significant changes in the time spent at the morphine-paired compartment. **p < 0.01 vs rats conditioned with morphine, *p < 0.05 vs rats conditioned with morphine (Tukey test); *CAFF* caffeine, *CGS* CGS 21680, *SCH* SCH 58261

**Fig. 3:**
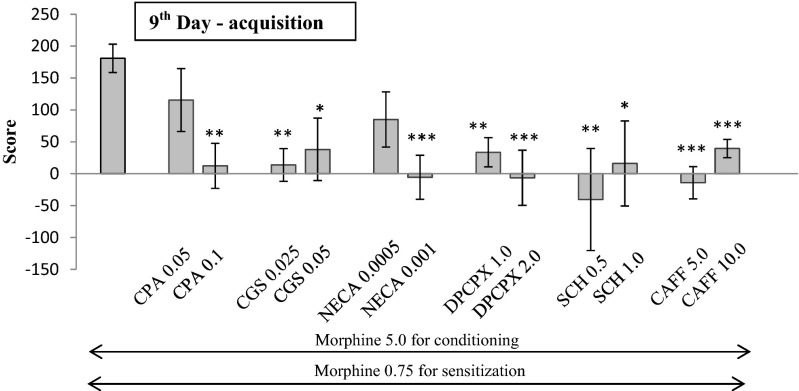
Influence of adenosine agonists and antagonists on the acquisition of sensitization to CPP in morphine-conditioned rats. Morphine sensitization was obtained by administration of priming dose of morphine (0.75 mg/kg, ip) in morphine-conditioned rats (5 mg/kg, ip). Adenosine ligands were injected three times, 20 min before each morphine injection (5.0 mg/kg), on 2nd–4th day. One-way ANOVA showed significant changes in the time spent at the morphine-paired compartment. ***p < 0.001 vs rats conditioned with morphine, **p < 0.01 vs rats conditioned with morphine, *p < 0.05 vs rats conditioned with morphine (Tukey test); *CAFF* caffeine, *CGS* CGS 21680, *SCH* SCH 58261

The two-way ANOVA was used for the effects of morphine and of the priming dose of morphine in the CPP test (Fig. 1.). One-way ANOVA was applied for the effects of adenosine drugs in morphine sensitization (Figs. 2 and 3.) and for statistical analysis of the locomotor activity of rats (Table 1). Post hoc comparisons were carried out by means of the Tukey test. The probability (*p*) value of 0.05 or less was considered as statistically significant. Each group of animals consisted of 9–10 rats.

**Table 1:** Effects of morphine and adenosine ligands on locomotor activity of rats

Locomotor activity of animals (Mean distance travelled [*m*])			
Drug	Acute dose	Drug combinations	
Saline	24.15 ± 2.3	Morphine 0.5 in sensitized rats	25.87 ± 2.1
Morphine 0.5	22.38 ± 3.7	Morphine 0.75 in sensitized rats	26.36 ± 3.1
Morphine 0.75	25.05 ± 1.6	CPA 0.1 + morphine 0.75 in sensitized rats	23.98 ± 3.6
Morphine 5.0	27.32 ± 4.0	CGS 0.05 + morphine 0.75 in sensitized rats	24.16 ± 4.2
CPA 0.1	22.11 ± 2.3	NECA 0.001 + morphine 0.75 in sensitized rats	26.74 ± 2.9
CGS 0.05	21.15 ± 3.1	DPCPX 2.0 + morphine 0.75 in sensitized rats	27.17 ± 4.3
NECA 0.001	22.02 ± 4.0	SCH 1.0 + morphine 0.75 in sensitized rats	29.79 ± 3.1
DPCPX 2.0	27.87 ± 3.2	CAFF 10.0 + morphine 0.75 in sensitized rats	29.87 ± 2.1
SCH 1.0	29.87 ± 2.6		
CAFF 10.0	30.77 ± 3.2		

There were not observed any alters in locomotor activity of rats after administration of all doses of morphine, adenosine ligands, and their combination

## Results

### Effects of morphine and adenosine ligands on locomotor activity of rats (Table 1)

There were not observed any alterations in locomotor activity of rats after administration of all doses of morphine, adenosine ligands, and their combination.

### Effects of morphine and priming dose of morphine in CPP test on rats (Fig. 1.)

Two-way ANOVA demonstrated some changes in behavior of the morphine preferring rats after the administration of the priming dose of morphine (0.5 and 0.75 mg/kg). After the application of morphine in dose of 0.75 mg/kg, a significant effect of drug (F1,32 = 19.32, *p* = 0.0001), day (F1,32 = 6.993, *p* = 0.0126), and interaction (F1,32 = 2,37 *p* = 0.0363) was observed in animals. An injection of morphine at a dose of 0.5 mg/kg as the priming dose induced a significant effect of the drug (F1,32 = 12.63, *p* = 0.0012) but not of the day (F1,32 = 3.407, *p* = 0.0742) and interaction (F1,32 = 0.241, *p* = 0.6268).

In the post hoc test, we demonstrated that an administration of morphine (5.0 mg/kg, ip) caused a significant increase in the time spent at the morphine-paired compartment, in comparison with saline administration (*p* < 0.01). A significant increase was also observed in the conditioning score after application of morphine in dose of 0.75 mg/kg as the priming dose—*p* < 0.01 in compared with saline challenged rats, but no significant changes were observed after the injection of 0.5 mg/kg of morphine as the priming dose. Based on those data, the dose of 0.5 mg/kg was selected as an ineffective dose for remaining experiments. In subsequent experiments, a higher dose of morphine—0.75 mg/kg—was used as the priming dose.

### Effects of adenosine ligands on the expression of sensitization to CPP in previously conditioned rats (Fig. 2.)

One-way ANOVA demonstrated that significant changes were observed only after the administration of adenosine A_1_ receptor ligands: agonist—CPA (F2,31 = 8.377, *p* = 0.0013) and antagonist—DPCPX (F2,32 = 5.009, *p* = 0.0133). Insignificant changes were evaluated after the administration of adenosine A_2A_ receptor ligands: agonist—CGS 21680 (F2,29 = 1.858, *p* = 0.1755) and antagonist—SCH (F_2,30_ = 2.82, *p* = 0.766) or non-selective A_1_/A_2A_ ligands: agonist—NECA (F2,31 = 2.247, *p* = 0.1238) and antagonist—caffeine (F2,34 = 0.4477, *p* = 0.643).

It was shown in the Tukey test that both doses of CPA significantly reduced (*p* < 0.01) the conditioning score on the 9th day of the experiment and both doses of DPCPX significantly increased the time spent at the morphine-paired compartment (*p* < 0.05). Other adenosine ligands did not produce any significant differences.

### Effects of adenosine ligands on the acquisition of sensitization to CPP in previously conditioned rats (Fig. 3.)

One-way ANOVA demonstrated significant changes after all the used adenosine ligands: CPA (F2,33 = 6.74, *p* = 0.0037), CGS 21680 (F2,37 = 7.826, *p* = 0.0016), NECA (F2,38 = 8.281, *p* = 0.0011), DPCPX (F2,33 = 11.49, *p* = 0.0001), SCH 58261 (F2,29 = 6.22, *p* = 0.0153), and caffeine (F2,35 = 22.87, *p* = 0.0001).

In the Tukey test, all the adenosine ligands (agonists and antagonists) significantly reduced the voluntary rat confinement in the morphine-paired compartment. Significant effects were observed after the administration of a higher dose of CPA (0.1 mg/kg)—(*p* < 0.01) and NECA (0.001 mg/kg)—(*p* < 0.001). Significant changes were also observed after an application of both doses of CGS 21680 (0.025 and 0.05 mg/kg)—*p* < 0.01 and *p* < 0.05, respectively; DPCPX (1.0 and 2.0 mg/kg)—*p* < 0.01 and *p* < 0.001, respectively; SCH 58261 (0.5 and 1.0 mg/kg)—*p* < 0.01 and *p* < 0.05, respectively; and caffeine (5.0 and 10.0 mg/kg)—*p* < 0.001.

## Discussion

In the presented experiment, we confirmed that the sensitization to CPP, induced by low doses of morphine, was developed in rats which were previously conditioned with morphine. But the major finding concerns an important role of the adenosinergic system, both in sensitization expression and acquisition.

In the first step of the experiments, we confirmed the results of other authors (Alaei and Hosseini 2007; Sahraei et al. 2007; Shippenberg et al. 2009). showing that morphine, given at a dose of 5.0 mg/kg and in a 3-day schedule, produced its rewarding effect, as expected. Our results are consistent with the literature data, which demonstrated a rewarding action of morphine at a dose range between 2.0 and 10.0 mg/kg (Leri and Franklin 2000; Lu et al. 2002; Sahraei et al. 2007). Therefore, in order to induce sensitization to CPP in rats, which were previously conditioned with morphine, a low dose of morphine (0.5 and 0.75 mg/kg) was administered after 5-day morphine treatment withdrawal (a method described by Sahraei at al. 2007). We observed that 0.75 mg/kg of morphine significantly increased in voluntary rat confinement periods at a morphine-paired compartment, which confirmed that sensitization to CPP was developed in the studied animals, while there was no such effect in the rats, treated with 0.5 mg/kg of morphine. Therefore, the dose of 0.75 mg/kg of morphine was used in further experiments, studying morphine-induced sensitization to CPP in previously morphine-conditioned rats.

Second, an involvement of the adenosinergic system was demonstrated in the morphine-induced sensitization to CPP. For this purpose, we used various adenosine receptor ligands (CPA, CGS 21680, NECA, DPCPX, SCH 58261, and caffeine), which are commonly used as pharmacological tools in various experimental protocols. All of them were used at low, ineffective doses, which was demonstrated in the locomotor activity test (see Table 1). These doses are commonly used in behavioral experiments (Karcz-Kubicha et al. 2003; Kopf et al. 1999; Munzar et al. 2002; Salem and Hope 1997).

We demonstrated that adenosine A_1_ receptors played an important role in the expression of the morphine-induced sensitization to CPP because A_1_ agonist—CPA—significantly reduced, while A_1_ antagonist—DPCPX—markedly increased the time, spent by rats at the preferred compartment. These results are in agreement with the results of other authors from their studied behavioral sensitization (Hobson et al. 2012; Knapp et al. 2001; Listos et al. 2011; Shimazoe et al. 2000). The adenosine A_2A_ receptors seem to be less important in expression of the sensitization to CPP in morphine-conditioned rats because CGS 21680 and SCH 58261 did not induce any significant effects in the studied rats. However, their role should not be completely excluded. In case of a higher dose of CGS 21680 and a lower dose of SCH 58261, the results were close to the statistical significance. Other authors demonstrated an inhibitory activity of A_2A_ receptors in the expression of behavioral sensitization in different models (Filip et al. 2006; Hobson et al. 2012; Knapp et al. 2001; Shimazoe et al. 2000). but those experiments were focused on sensitization to cocaine and methamphetamine. There are no literature data to show the effects of adenosine A_2A_ receptors in morphine sensitization expression. The mechanism of action of cocaine or methamphetamine is different from that of morphine, which may explain the behavioral discrepancies between the effects of A_2A_ receptors in cocaine/methamphetamine and morphine sensitization models. Interestingly enough, a simultaneous stimulation or blockade of both A_1_ and A_2A_ adenosine receptors by NECA or caffeine, respectively, did not induce any effect in the studied rats, either. Lack of the effect after administration of NECA and caffeine in the expression of sensitization to CPP in morphine-conditioned rats seems to be associated with interactions between adenosine receptors. Cunha et al. (1994) described an interaction between A_1_ and A_2A_ receptors. According to that, in some circumstances (high frequency stimulation), A_2A_ receptors are able to decrease the activity of A_1_ receptors. A few reports also document the existence of interactions between adenosine A_2B_/A_3_ and A_1_ receptors. The administration of selective adenosine A_2B_ agonist (BAY 60-6583) attenuated the activity of DPCPX (Gonçalves et al. 2015). and A_3_ receptor activation reduced the inhibitory action of adenosine A_1_ receptors in the hippocampus (Dunwiddie et al. 1997). Sebastiao and Ribeiro (2009) precisely described that phospholipase C-coupled response of metabotropic A_2A_, A_2B_, and A_3_ receptors may be involved in inhibition of presynaptic A_1_ receptors in nerve terminals. As literature data report, DPCPX has an affinity mainly for A_1_ receptors but it may also act on A_2B_ receptors (Fredholm et al. 2001, 2011). While NECA, as non-selective adenosine receptor agonist, is able to produce the effects mainly by stimulation of A_1_ and A_2A_ receptors but also by A_2B_ and A_3_ receptors (Volpini et al. 2003; Fredholm et al. 2011). In our experiments, we used low doses of all adenosine compounds, and involvement of adenosine A_2B_ and A_3_ receptors seems to be insignificant for the obtained results; however, the lack effect of NECA in morphine expression may be explaind by interaction between adenosine receptors.

Furthermore, the effect of adenosine ligands in the acquisition of sensitization to CPP in rats was a fairly interesting observation. All the adenosine ligands, administered on the 2nd–the 4th day, significantly inhibited the acquisition of sensitization to CPP in rats. The inhibitory effect of all adenosine ligands in acquisition is intriguing, but not surprising. There are scientific reports, showing a similar pattern of the ligands’ activity. For example, A_1_ agonists and antagonists are able to reduce hyperalgesia, or A_2A_ agonists and antagonists decrease neuronal death by reducing of neurotransmitter release (for ref. see Stone et al. 2009). We suppose that physiological relationships between adenosine and dopamine receptors influence glutamate release from the presynaptic terminals, where A_1_ and A_2A_ receptors are also strongly expressed. It is worth noting that other authors demonstrated some attenuation of nicotine- (Castañé et al. 2006). amphetamine- (Bastia et al. 2005). and cocaine- (Soria et al. 2006) induced rewarding effects in mice with lacking A_2A_ receptors. Weisberg and Kaplan (1999) demonstrated that A_1_ antagonist attenuated the development of morphine sensitization. On the other hand, Brown et al. (2009) showed that A_2A_ receptor was not necessary for the development of morphine sensitization.

The participation of adenosinergic system in behavioral sensitization has been already described in our previous papers. Then, we showed that CPA, CGS 21680, and NECA were able to inhibit the acquisition of morphine-seeking behavior in mice, observed as the sensitization to the locomotor activity (Listos et al. 2011). In another one, CGS 21680 and NECA, but not CPA, reduced the hypersensitivity to acute dose of morphine administered during morphine withdrawal period (Listos et al. 2008). Thus, both papers presented the beneficial role of adenosine receptor agonists in various tests reflected the relapse to drug use. At present, in rats, we significantly extend this knowledge, because the effect of adenosine agonists and antagonists was studied in, both, expression and acquisition of sensitization to CPP. This test reflected the sensitization to morphine rewarding effect. In this context, the participation of adenosinergic system was examined for the first time. In all these studies, we comprehensively showed an important role of adenosinergic system in various aspects of morphine sensitization.

Considering potential mechanisms, which may underlie the obtained results, several options should be taken into account. First, the distribution of adenosine A_1_ and A_2A_ receptors in the central nervous system and their adaptive changes after chronic opioid treatment should become subject of analysis. Physiologically, A_1_ receptors are abundant in the whole brain, while A_2A_ receptors are located mainly on striatopallidal γ-aminobutyric acid (GABA)ergic neurons, in the olfactory bulb and the hippocampus. The activity of A_1_ receptors is associated with the reduced release of different neurotransmitters in the central nervous system and, in this way, with suppressing neuronal activity in the brain (Sebastiao and Ribeiro 2009). while A_2A_ receptors are involved in various interactions with other receptors, such as glutamatergic, dopamine D_2_, or cannabinoid CB_1_ receptors (for ref. see Sebastiao and Ribeiro 2009). However, after chronic morphine treatment, some adaptive changes may develop, mainly in adenosine A_1_ receptors. For example, a significant increase was observed in the number of A_1_ receptors (Kaplan et al. 1994). in the amount of adenosine transporters (Kaplan and Leite-Morris 1997) and in adenosine sensitivity in nucleus accumbens (Brundege and Williams 2002). On the contrary, a chronic opioid treatment does not seem to affect the A_2A_ receptor level because the number of A_2A_ receptors in the striatum remained unaltered after a chronic morphine treatment in mice (Kaplan et al. 1994).

Second, apart from the neuroadaptive changes in the adenosinergic system, other mechanisms seem to be engaged. Several findings demonstrate that, during morphine-induced sensitization, alterations in dopaminergic and glutamatergic receptors may occur within some brain areas (ventral tegmental area, nucleus accumbens, prefrontal cortex, amygdala, and hippocampus) (Robinson and Berridge 2008; Vanderschuren and Kalivas 2000; Vanderschuren and Pierce 2010; Wolf 2003). The expression of sensitization is associated with increased extracellular dopamine levels, the supersensitivity of D_1_ receptors in the striatum (Tjon et al. 1994, 1997). and elevated dopamine levels in nucleus accumbens (Kalivas and Duffy 1987; Spanagel and Shippenberg 1993; Spanagel et al. 1993) in sensitized animals. Otherwise, the development of morphine-induced sensitization is more related to the glutamatergic system and the ventral tegmental area; the antagonists of both types of glutamate receptors—NMDA (MK-801 and CGS 19755) and AMPA (LY293558)—were able to prevent the morphine sensitization process (Jeziorski et al. 1994; Carlezon et al. 1999, respectively). It is likely that the neuroadaptive changes in dopamine receptors (expression) as well as in glutamate receptors (acquisition) could have affected the results in our study, especially that both dopamine and glutamate receptors are closely linked to adenosine receptors and the interactions between dopamine–adenosine receptors and glutamate–adenosine receptors (for ref. see Fredholm 2001; Sebastião and Ribeiro 2009) are well described.

Considering all the connections between adenosine and other receptors in the central nervous system, it may be suggested that the involvement of A_1_ ligands in the expression of sensitization to CPP might have been associated with a morphine-induced increase in the number of adenosine A_1_ receptors and in a more expressed effect on D_1_ receptors. On the other hand, the inhibitory effect of both adenosine agonists and antagonists in the acquisition of sensitization to CPP seems to be associated with neuroadaptive changes within the glutamatergic system, the role of which in behavioral sensitization is undisputed (Vanderschuren and Kalivas 2000; Vanderschuren and Pierce 2010).

In conclusion, behavioral sensitization is a major characteristic of drug addiction and could be used to study the effects of addictive, abused drugs. In humans, drug-seeking behaviors are strongly manifested even after long-term cessation periods and are responsible for drug relapse. It is, therefore, extremely important to explore all the mechanisms associated with sensitization. In the presented findings, the sensitization to the CPP was confirmed in rats, after the priming dose of morphine, the rats having previously been conditioned with morphine. We also observed that adenosine A_1_ receptor played an important role in the expression of the morphine-induced sensitization to CPP. Finally, we indicated that both stimulation and blockade of A_1_ and/or A_2A_ receptors inhibited the acquisition of the morphine-induced sensitization to CPP in rats. The obtained results strongly support the significance of the adenosinergic system, both in the expression and acquisition of sensitization to CPP. They seem to be important for the identification of connections in the central nervous system which can help finding new strategies to attenuate rewarding action of morphine.
